# The Effects of Instruction Manipulation on Motor Performance Following Action Observation

**DOI:** 10.3389/fnhum.2020.00033

**Published:** 2020-03-06

**Authors:** Silvi Frenkel-Toledo, Moshe Einat, Zvi Kozol

**Affiliations:** ^1^Department of Physical Therapy, School of Health Sciences, Ariel University, Ariel, Israel; ^2^Department of Neurological Rehabilitation, Loewenstein Hospital, Raanana, Israel; ^3^Department of Electrical and Electronic Engineering, Ariel University, Ariel, Israel

**Keywords:** action observation, instruction, motor performance, attention, motor imagery

## Abstract

The effects of action observation (AO) on motor performance can be modulated by instruction. The effects of two top-down aspects of the instruction on motor performance have not been fully resolved: those related to attention to the observed task and the incorporation of motor imagery (MI) during AO. In addition, the immediate vs. 24-h retention test effects of those instruction’s aspects are yet to be elucidated. Forty-eight healthy subjects were randomly instructed to: (1) observe reaching movement (RM) sequences toward five lighted units with the intention of reproducing the same sequence as fast and as accurate as possible (Intentional + Attentional group; AO+At); (2) observe the RMs sequence with the intention of reproducing the same sequence as fast and as accurate as possible and simultaneously to the observation to imagine performing the RMs (Intentional + attentional + MI group; AO+At+MI); and (3) observe the RMs sequence (Passive AO group). Subjects’ performance was tested before and immediately after the AO and retested after 24 h. During each of the pretest, posttest, and retest, the subject performed RMs toward the units that were activated in the same order as the observed sequence. Occasionally, the sequence order was changed by beginning the sequence with a different activated unit. The outcome measures were: averaged response time of the RMs during the sequences, difference between the response time of the unexpected and expected RMs and percent of failures to reach the target within 1 s. The averaged response time and the difference between the response time of the unexpected and expected RMs were improved in all groups at posttest compared to pretest, regardless of instruction. Averaged response time was improved in the retest compared to the posttest only in the Passive AO group. The percent of failures across groups was higher in pretest compared to retest. Our findings suggest that manipulating top-down aspects of instruction by adding attention and MI to AO in an RM sequence task does not improve subsequent performance more than passive observation. Off-line learning of the sequence in the retention test was improved in comparison to posttest following passive observation only.

## Introduction

Observing the actions of others can act as a type of training, which may improve motor performance (Heyes and Foster, 2002; Bird and Heyes, 2005; Mattar and Gribble, 2005; Porro et al., 2007). This has been demonstrated among healthy individuals in various motor tasks such as sports activities (Weeks and Anderson, 2000; Horn et al., 2007), novel tasks (Mattar and Gribble, 2005), and force tasks (Porro et al., 2007). Positive behavioral effects of action observation (AO) training have been shown also in neurological conditions such as stroke (Ertelt et al., 2007; Sugg et al., 2015; Patel, 2017; Peng et al., 2019), Parkinson’s disease (Pelosin et al., 2010, 2013; Giorgi et al., 2018), cerebral palsy (Buccino et al., 2012; Sgandurra et al., 2013), and orthopedic conditions (Bellelli et al., 2010; Park et al., 2014).

The effects of AO on motor performance were proposed to be mediated *via* the human mirror neuron system (MNS) that is activated during both execution of a motor act and observation of that act performed by others (Rizzolatti and Craighero, 2004; Buccino, 2014). This dual activation was first demonstrated in cortical neurons of macaque monkeys termed “mirror neurons” (di Pellegrino et al., 1992; Fogassi et al., 2005; Rizzolatti and Sinigaglia, 2010, 2016). Later, a human analog of the MNS was suggested on the basis of functional brain imaging (Buccino et al., 2004; Fabbri-Destro and Rizzolatti, 2008; Morin and Grèzes, 2008), transcranial magnetic stimulation (Fadiga et al., 1995), single-unit recording (Mukamel et al., 2010), magnetoencephalography (Hari, 2006), and electroencephalography (EEG; Muthukumaraswamy et al., 2004; Pineda, 2005).

The extent of the AO effects on motor performance can be modulated by several factors such as the observed model type (Rohbanfard and Proteau, 2011), advanced information about the demonstration quality (Andrieux and Proteau, 2016), viewpoint (Watanabe and Higuchi, 2016), and visual guidance (D’Innocenzo et al., 2016). With respect to the observed model type, participants who were required to observe a four-segment timing task performed by a novice, expert, or both novice and expert models outperformed a control group on both total movement time and intermediate time of each segment in the immediate retention and transfer tests (Rohbanfard and Proteau, 2011). Using a similar task, another study (Andrieux and Proteau, 2016) found that learning was optimized when knowledge about the quality and characteristics of the demonstration (total movement time and intermediate time) was presented before each demonstration as opposed to after each demonstration. Learning, reflected by shorter response latencies and fewer errors, was also shown to be optimized following observation from a first-person perspective in contrast to a third-person perspective in another timing task—index finger lifting from a resting position (Watanabe and Higuchi, 2016). In addition, improvement of golf swing execution (rated scores) was found at posttest and 1 week later in a retention test among participants who spent significantly more time looking at cued areas on video clips (D’Innocenzo et al., 2016). In AO literature, the question concerning the most effective instruction for optimizing the motor performance of an observer remains open.

The effect of top-down aspect of instruction, which relates to the incorporation of motor imagery (MI; i.e., imagining the execution of an action without physically performing it; Jeannerod, 1994; Hardwick et al., 2018) during AO on the subsequent motor performance has not been fully resolved. From a neural point of view, a partial neural activity overlap was found between AO, MI, and movement execution (Burianová et al., 2013; Kraeutner et al., 2014; Duann and Chiou, 2016; Hardwick et al., 2018; Solomon et al., 2019). According to a recent meta-analysis (Hardwick et al., 2018), AO and MI recruited similar premotor-parietal cortical networks. Furthermore, while MI and movement execution recruited a similar subcortical network, AO did not consistently recruit subcortical areas. Specifically, in the Hardwick et al. (2018) meta-analysis, contrast analyses, which identify regions more consistently implicated with one of two compared tasks, showed that movement execution, in comparison to MI, was associated with the recruitment of the left putamen and right cerebellum. Additional differences in brain regions involved in MI vs. movement execution were found by Burianová et al. (2013). They showed that MI exclusively activated areas—the bilateral occipital gyrus, left inferior parietal lobule (IPL), parahippocampus, right superior temporal gyrus, and superior frontal gyrus—that are part of a circuitry important for visuospatial imagery, the processing and remembering of visual scenes, and the representation of three-dimensional space (Epstein and Kanwisher, 1998; Mullaly and Maguire, 2011). Also, EEG studies showed partially resembled event-related desynchronization patterns in movement execution compared to MI and AO (Kraeutner et al., 2014; Duann and Chiou, 2016; Solomon et al., 2019). For example, the magnitude of event-related desynchronization in sensorimotor regions was significantly greater in movement execution than MI during both the preparatory and performance phases (Solomon et al., 2019).

The effect of the additional top-down aspect of instruction, which relates to an increased attention to the observed task during AO on the subsequent motor performance, has also not been fully resolved. Several studies found that incorporating attention into AO enhanced the effects of AO on the subsequent motor performance (Janelle et al., 2003; Badets et al., 2006; Bach et al., 2007; Gowen et al., 2010; Hayes et al., 2014; Bek et al., 2016). For example, adding verbal and visual cues enhanced learning of soccer kicking by observation (Janelle et al., 2003). Coding of biological motion kinematics was augmented when instructing observers to attend to movement trajectory during observational learning of human movement sequences depicted by a mouse cursor and was attenuated when attentional resources were divided (Hayes et al., 2014). On the other hand, Mattar and Gribble (2005) concluded that observational learning is not based on attention-explicit conscious strategies; instead, it depends on the implicit engagement of neural systems dedicated to movement planning and control. This was demonstrated by showing that an arithmetic distracting task during AO did not affect subsequent performance when compared to observation without such a distracting task (see also Vinter and Perruchet, 2002). Indeed, a recent meta-analysis of nonhuman primates (Loonis et al., 2017) showed that explicit vs. implicit learning engages different neural mechanisms that rely on different patterns of oscillatory synchrony. Also, from the behavioral point of view, it was suggested that the implicit motor learning of golf-putting could be beneficial for children with a low motor ability, in contrast to children with a high motor ability, who tend to benefit from explicit motor learning (Maxwell et al., 2017).

Only a few studies have investigated whether engaging in MI during AO affects the subsequent performance (Eaves et al., 2014, 2016a; Bek et al., 2016). MI training was found to improve motor skills (Lotze and Halsband, 2006; Mizuguchi and Kanosue, 2017). Combining MI training with physical practice has generally been found to be more effective than physical practice alone (e.g., Allami et al., 2008). Engaging in MI during AO enhanced the magnitude of imitation bias relative to passive AO (Eaves et al., 2014) and pure MI (Eaves et al., 2016a). Similarly, neuroimaging studies have revealed stronger activations during concurrent AO and MI than during AO alone across motor areas including regions of premotor cortex and inferior parietal cortex (Nedelko et al., 2012). Increased event-related desynchronization in theta (4–8 Hz), alpha (8–13 Hz), and beta (13–25 Hz) frequency bands in sensorimotor areas during AO combined with MI compared to AO alone has also been found using EEG (Berends et al., 2013), and conflicting MI with AO abolished event-related desynchronization pattern effects (Eaves et al., 2016a; Sun et al., 2016).

To our knowledge, only two studies directly compared the effects of AO+Attention (AO+At) vs. AO+MI on subsequent motor performance (Bek et al., 2016; Di Rienzo et al., 2019). In the study by Bek et al. (2016), healthy participants were asked to observe and imitate (i.e., intentional observation) human hand movement sequences, while movement kinematics were recorded. Participants were instructed to either imagine performing the observed movement or attend closely to the characteristics of the movement, or received no further instructions (control). Kinematics of the imitated movements were similarly modulated in the MI and Attention groups, being closer in duration, peak velocity, and amplitude to the observed model compared with controls. In a study by Di Rienzo et al. (2019), healthy participants were asked to carefully observe an athlete performing a maximal voluntary contraction while attending to specific characteristics and refraining from MI or imagining themselves attempting to lift the platform while feeling the contraction. The control group passively watched a video documentary about shooting baskets (basketball). Here as well, no superiority effect of AO+MI on muscle function was found when compared to the AO+Attention, while both AO+Attention and AO+MI outperformed the control condition in terms of total force. Simultaneous MI and AO were found to significantly enhance corticomotor excitability in comparison to pure AO (Wright et al., 2016; Cengiz et al., 2018). Being instructed to observe passively, or with the intent to imitate the observed movement, or while simultaneously and actively imagining self-performance of the movement during observation facilitated corticospinal excitability to a greater extent than observation of a static hand. In addition, corticospinal excitability was facilitated to a greater extent during combined observation and imagery as compared to passive observation (Wright et al., 2016).

Given the potential application of AO as a neurorehabilitation tool, one key step for maximizing the impact of AO therapy is to define the optimal instruction that can lead to the best subsequent motor performance. The effects of directed attention and incorporated MI to the observed action on behavioral outcomes are limited. In the two relevant studies mentioned above (Bek et al., 2016; Di Rienzo et al., 2019), only the immediate effects were investigated. Whereas AO was found to trigger consolidation processes that lead to a stabilization of the new motor skill and off-line gains (Trempe et al., 2011; Hesseg et al., 2016), the delayed retest effects as a function of instruction manipulation (relating to attention and MI during AO) is yet to be elucidated. In addition, although Bek et al. (2016) and Di Rienzo et al. (2019) studied the effects of AO+Attention or AO+MI, they did not investigate the combined effect of AO+Attention+MI. Here, we compared the immediate and 24-h retention test effects of combined intentional AO+Attention, as well as intentional AO+Attention+MI and non-intentional observation (passive AO). Since passive AO was found to induce incidental implicit learning (Mattar and Gribble, 2005), we also attempted to elucidate—for the first time, to the best of our knowledge—whether adding attention and MI to the instruction would outperform learning that does not include explicit instruction about engaging in learning.

We hypothesized that: (1) subsequent performance will improve following passive AO due to implicit learning (Mattar and Gribble, 2005); (2) subsequent performance will improve to a greater extent following simultaneous engagement in attention and imagination of the observed task than after non-intentional passive AO. This is based on previous evidence concerning the positive effects on subsequent motor performance of attending to or imagining the observed task (Janelle et al., 2003; Badets et al., 2006; Bach et al., 2007; Gowen et al., 2010; Hayes et al., 2014; Bek et al., 2016), together with neurophysiological evidence for significantly greater cortico-motor activity during AO+MI as compared to AO alone (Nedelko et al., 2012; Taube et al., 2014; Eaves et al., 2016a; Hardwick et al., 2018); (3) subsequent performance will improve to a similar extent following simultaneous engagement in attention and imagination of the observed task, given that a direct comparison between AO+MI and AO+attention indicated a similar effect on kinematics and the force of the imitated movements (Bek et al., 2016; Di Rienzo et al., 2019); and (4) AO-related behavioral effects will be reflected in both averaged response time and segmental response time of reaching movement (RM) toward a specific direction. Since total sequential movement time and its specific segmental intermediate time improved following AO (Rohbanfard and Proteau, 2011) and hand trajectories improved more when tested in the same environment than in a different one (opposite direction; Mattar and Gribble, 2005), we chose to measure both the average response times of all movements and the difference between the response time of an unexpected and expected movement (toward the opposite and expected direction; respectively) toward a specific direction. Given the lack of relevant literature, we were unable, at this stage, to make a hypothesis about differences in 24-h retention test effects subsequent to the different instructions.

## Materials and Methods

### Participants

Forty-eight subjects (26 women, 22 men; aged 24 ± 2 years) participated in the study. In all subjects, the dominant right arm was tested. Participants were included if they were right-hand dominant and were healthy according to their report. They were excluded if they had musculoskeletal or neurological deficits interfering with task performance (proper reaching performance in sitting). Participants signed an informed consent form approved by the Ethics Committee of Ariel University. Sixteen participants were assigned to each group. One participant had to be excluded from the analysis due to technical problems. Hence, the reported results are based on 47 subjects.

### Experimental Procedures and Data Recording

**Figure 1:**
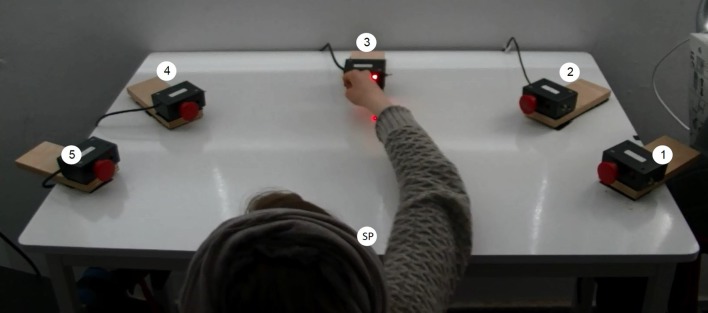
General setup. SP, starting position, where right fist was placed before reaching toward the lighted unit.

Apparatus used in tests (pretest, posttest, and retest): the custom made testing device was set up on a rectangular table with a smooth laminated tabletop of 105 cm × 80 cm and adjustable height. Five switch-led units of 5 cm × 8 cm × 5 cm, each composed of a large push-button switch and a red light-emitting diode (LED), attached to the tabletop in a 45-cm radius half circle, successively numbered from 1 to 5 (Figure 1). The system was operated by a desktop computer, interfaced with a data acquisition card of LabVIEW software. The algorithm allowed parameters selection of LED activation (illumination) sequence, duration of RM, delay between RMs, and number of RM repetitions. An activation of a specific unit LED was a cue for the subject to reach toward that unit and press the push-button switch. Reaching toward the switch of an activated unit deactivated it, and the response time, between the activated and deactivated LED, was recorded. The initial testing position of the subjects was sitting on a chair with a solid back support in front of a table, hips and knees flexed 90°. At the starting position, the right fist of the participants was placed on the edge of the table in front of their chest (parallel to switch 3), so that they could reach and touch switch 3 with their third right metacarpal.

#### Procedure and Data Analysis

Subjects participated in a session that included familiarization practice, pretest, video clip observation, posttest, and retention test after 24 h. Subjects were randomly assigned to one of three groups that received the following different instructions prior to video clip observation of RM sequence: (1) Intentional + attentional group (AO+At): “Observe carefully the RM sequence and learn the sequence. After the observation, you will be asked to perform the same sequence as fast and as accurate as possible”; (2) Intentional + attentional + MI group (AO+At+MI): “Observe carefully the RM sequence to learn the sequence and simultaneously to the observation imagine that you are performing the RM with your right upper limb”; and (3) Passive observation (Passive AO): “Observe the RM sequence.” All subjects were asked to avoid moving during the video clip observation. The observed sequence (7 s each) in the video clip consisted of six RMs toward the units in the order of 1, 4, 3, 5, 4, 2 (averaged response time of the observed RM: 550.47 ± 35 ms). The observed RM sequence was performed by a young healthy female (23 years), with her dominant right hand on the same apparatus used in the tests. Each subject observed 60 sequences (360 RMs) from an egocentric viewpoint as it was found to be more effective than an allocentric viewpoint (Watanabe and Higuchi, 2016; Angelini et al., 2018), with a rest period of 30 s after observing 20 and 40 sequences.

At the beginning of the experiment, subjects performed a familiarization practice of 30 randomized RMs, which consisted of reaching with their right hand toward the activated unit, touching the unit-related switch as fast as possible, and returning the hand to the starting position until the next unit was activated. During each pretest, posttest, and retest, the subject performed RMs toward the units that were activated in the same order as the observed sequence (1, 4, 3, 5, 4, 2) and with an activation duration and delay of 1 s. If the subject did not reach toward the activated unit and touched the unit-related switch within 1 s, the trial was considered a “fail” and was not included in the averaged response time. During each pretest, posttest, and retest, the subjects executed 20 sequences (i.e., 120 RMs/trials); five sequences constituted a block. The subjects rested for 30 s after each block. In the fifth, third, fourth, and second sequence of the first, second, third, and fourth blocks, respectively, the sequence order of the test changed unexpectedly and began with unit 5 instead of unit 1; that is, 5, 4, 3, 5, 4, 2 instead of 1, 4, 3, 5, 4, 2. The averaged response times of the RMs toward all the targets and toward unit 5 during the regular sequence and the unexpected changed sequence were recorded.

Outcome measures were made by averaging the response time of all the RMs during the sequences (termed Seq, in milliseconds), the difference between the response time of the unexpected and expected RMs toward unit 5 (termed Delta, in milliseconds) and the percent of fails (referred to as “Fail”) was calculated for each block as (number of fails/30 trials)*100. Since the time limit for each RM was 1 s, and for each pretest, posttest, and retest Delta was averaged across four trials only, specifically for Delta, the value 1,001 ms was given for a RM toward the unexpected unit 5 that was not executed within 1 s (for a similar approach, see Bogard et al., 2009; Fritz et al., 2009). Improved motor performance was indicated by a shorter response time (lower Seq), larger time differences (i.e., a larger discrepancy) between the unexpected and expected RMs toward unit 5 (higher Delta), and less failures.

### Statistical Analysis

Age and gender were compared between groups (AO+At, *n* = 16; AO+At+MI, *n* = 16; Passive AO, *n* = 15) using Kruskal–Wallis (as age was not normally distributed) and chi-square tests, respectively.

The two main outcomes, Seq and Delta, were normally distributed. The third outcome, Fail, did not distribute normally; therefore, we used a log transform of the original value + 1 (the addition of the value of 1 is related to the fact that some subjects had zero failures). The differences between groups with respect to each of the main outcomes in the pretest were investigated using one-way ANOVA with Bonferroni correction for multiple comparisons.

The effects of instruction and time on the main outcomes (Seq, Delta and Fail) were investigated using mixed ANOVA with time (pretest, posttest, retest) as within-subject factor and group (AO+At, AO+At+MI, Passive AO) as the between-subject factor with Bonferroni correction for multiple comparisons. The Greenhouse–Geisser Epsilon (G-GE) was used to correct the degrees of freedom when the Mauchly’s test of sphericity was significant.

All tests were done using SPSS (version 25.0) with initial significance levels of *p* < 0.05.

## Results

Age (AO+At: 24 ± 2 years; AO+At+MI: 25 ± 3 years; Passive AO: 25 ± 2 years) and gender (AO+At: nine women; AO+At+MI: eight women; Passive AO: eight women) were matched between groups.

Mean values of Seq (in milliseconds), Delta (in milliseconds), and Fail (in percent) by group and time are shown in Table 1. In Table 1, we reported the original values of the Fail (mean ± SD) for purposes of clarity. Seq, Delta, and Fail were matched between groups in the pretest (*F*_(2,44)_ = 0.315, *p* = 0.731; *F*_(2,44)_ = 1.133, *p* = 0.331; and *F*_(2,44)_ = 1.764, *p* = 0.183, respectively).

**Table 1:** Means and standard deviations of Seq, Delta, and Fail for instruction groups in time points.

Variable	AO+At (*n* = 16)			AO+At+MI (*n* = 15)			Passive AO (*n* = 16)		
	Pretest	Post test	Retest	Pre test	Post test	Retest	Pre test	Post test	Retest
Seq (ms)	625.48 ± 92.20	513.83 ± 110.15	485.20 ± 121.92	644.05 ± 86.17	567.11 ± 62.45	574.34 ± 65.55	647.76 ± 75.46	587.86 ± 107.49	535.12 ± 123.80
Delta (ms)	47.13 ± 67.52	291.61 ± 116.39	228.54 ± 188.62	54.32 ± 45.10	249.96 ± 141.58	168.32 ± 100.51	85.46 ± 103.26	232.84 ± 170.19	201.88 ± 140.72
Fail (%)	5.10 ± 4.54	2.71 ± 2.01	1.98 ± 2.33	2.00 ± 2.31	1.17 ± 1.47	0.83 ± 0.94	3.65 ± 5.05	2.71 ± 2.93	1.61 ± 1.23

*AO+At, intentional + attentional action observation; AO+At+MI, intentional + attentional action observation combined with simultaneous motor imagery; Passive AO, passive action observation*.

### Seq (ms)

**Figure 2:**
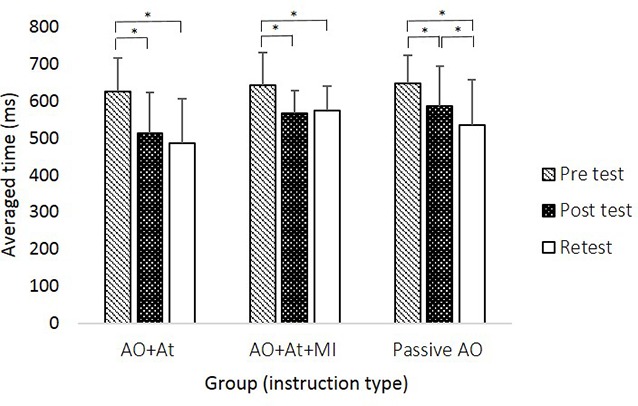
Averaged time (in milliseconds) of reaching movements (RMs) during all sequences in each group at the different time points. AO+At, intentional + attentional action observation; AO+At+MI, intentional + attentional action observation combined with simultaneous MI; Passive AO, passive action observation. Asterisks denote a significant difference (pBonferroni < 0.05).

A main effect of Time (*F*_(2,88)_ = 59.906; *p* < 0.001; partial *η*^2^ = 0.56; observed power = 1.00) showed that across groups, averaged time was longer in pretest (638.99 ± 83.59 ms) compared to posttest (556.03 ± 99.61 ms, pBonferroni < 0.001) and retest (530.64 ± 111.80 ms; pBonferroni < 0.001) and was significantly longer in posttest compared to retest (pBonferroni = 0.024). The interaction of Group × Time (*F*_(4,88)_ = 2.843, *p* = 0.029; partial *η*^2^ = 0.11; observed power = 0.75) showed that only for the Passive AO group, averaged time was decreased significantly more in retest compared to posttest (retest: 535.12 ± 123.80 ms, posttest: 587.86 ± 107.49 ms, pBonferroni < 0.001), whereas in the AO+At (posttest: 513.83 ± 110.15 ms, retest: 485.20 ± 121.92 ms, pBonferroni = 0.174) and AO+At+MI groups (posttest: 567.11 ± 62.45 ms, retest: 574.34 ± 65.55 ms, pBonferroni = 1.000), it did not differ (Figure 2).

### Delta (ms)

**Figure 3:**
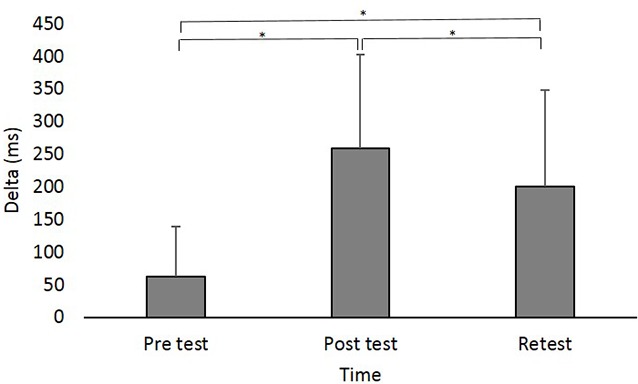
Delta (in milliseconds) at the different time points. Asterisks denote a significant difference (pBonferroni < 0.05). Main effect of Time (collpased across the groups) is presented because the interaction Group × Time was not significant.

A main effect of Time (*F*_(2,88)_ = 42.831; *p* < 0.001; partial *η*^2^ = 0.49; observed power = 1.00) showed that across groups, Delta was significantly shorter in pretest (62.47 ± 76.62 ms) compared to posttest (258.31 ± 143.52 ms; pBonferroni < 0.001) and retest (200.25 ± 147.46 ms; pBonferroni < 0.001) and was shorter in retest compared to posttest (pBonferroni = 0.024; Figure 3).

### Fail

**Figure 4:**
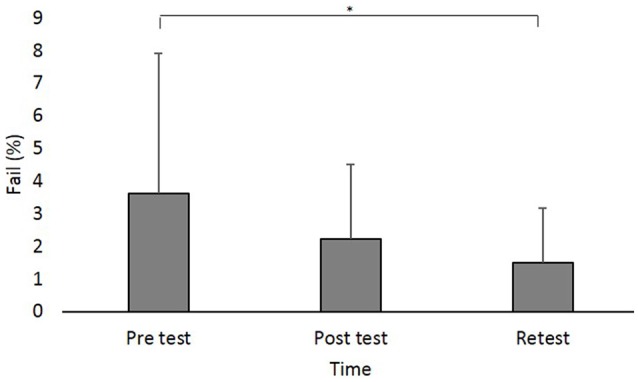
Fail (in percent) at the different time points. Asterisks denote a significant difference (pBonferroni < 0.05). Main effect of Time (collpased across the groups) is presented because the interaction Group × Time was not significant.

A main effect of Time (*F*_(2,88)_ = 8.642; *p* < 0.001; partial *η*^2^ = 0.16, observed power = 0.96) showed that across groups, the percent of Fails was significantly higher in pretest (log values: 0.50 ± 0.37, original values: 3.62% ± 4.28%) compared to retest (log values: 0.31 ± 0.27, original values: 1.49% ± 1.67%; pBonferroni = 0.003; Figure 4).

No other significant effects were observed. All effects are shown in Table 2.

**Table 2:** Results of mixed ANOVA with time (pretest, posttest, retest) as the within-subject factor and group (AO+At, AO+At+MI, Passive AO) as the between-subject factor for Seq, Delta, and Fail.

Variable	Main effects and interactions			*Post hoc* comparisons	
	Time	Group	Time × Group	Time	Time × Group
Seq (ms)	*F*_(2,88)_ = 59.906, *p* < 0.001, partial *η*^2^ = 0.56, observed power = 1.00	*F*_(2,44)_ = 1.816, *p* = 0.175, partial *η*^2^ = 0.08, observed power = 0.36	*F*_(4,88)_ = 2.843, *p* = 0.029, partial *η*^2^ = 0.11, observed power = 0.75	Pretest (longer) vs. posttest, pBonferroni < 0.001; Pretest (longer) vs. retest, pBonferroni < 0.001; Posttest (longer) vs. retest, pBonferroni = 0.024	Passive AO group: Pretest (longer) vs. posttest, pBonferroni < 0.007; Pretest (longer) vs. retest, pBonferroni < 0.001; Posttest (longer) vs. retest, pBonferroni < 0.001
					AO+At group: Pretest (longer) vs. posttest, pBonferroni = 0.001; Pretest (longer) vs. retest, pBonferroni < 0.001; Posttest vs. retest, pBonferroni = 0.174
					AO+At+MI group: Pretest (longer) vs. posttest, pBonferroni < 0.001; Pretest (longer) vs. retest, pBonferroni = 0.009; Posttest (longer) vs. retest, pBonferroni = 1.000
Delta (ms)	*F*_(2,88)_ = 42.831; *p* < 0.001, partial *η*^2^ = 0.49, observed power = 1.00	*F*_(2,44)_ = 0.434, *p* = 0.651, partial *η*^2^ = 0.02, observed power = 0.12	*F*_(4,88)_ = 1.070, *p* = 0.376, partial *η*^2^ = 0.05, observed power = 0.32	Pretest (shorter) vs. posttest, pBonferroni < 0.001; Pretest (shorter) vs. retest, pBonferroni < 0.001; Posttest (longer) vs. retest, pBonferroni = 0.024	
Fail (%)	*F*_(2,88)_ = 8.642, *p* < 0.001, partial *η*^2^ = 0.16, observed power = 0.96	*F*_(2,44)_ = 3.008, *p* = 0.06, partial *η*^2^ = 0.12, observed power = 0.55	*F*_(4,88)_ = 0.512, *p* = 0.727, partial *η*^2^ = 0.02, observed power = 0.17	Pretest vs. posttest, pBonferroni = 0.062; Pretest (more) vs. retest; pBonferroni = 0.003; Posttest vs. retest, pBonferroni = 0.102	

*Post hoc comparisons are mentioned only for significant main and interaction effects. Original values (not log values) of Fail are presented for clarity*.

## Discussion

The goal of the study was to elucidate the immediate and 24-h retention test effects of instruction on the subsequent motor performance of the observer. The performance of RM sequence was compared between healthy subjects who were asked simply to observe the sequence (Passive AO group) or were explicitly instructed that they would be required to execute the observed sequence as fast and as accurately as possible while attending closely to the observed sequence (AO+At group) or imagining simultaneously with the attended observation that they are performing the sequence (AO+At+MI group).

Averaged response time of the RMs sequence (Seq, in milliseconds) and the difference between the response time of the unexpected and expected RMs toward unit 5 (Delta, in milliseconds) were similarly improved in posttest and retest compared to pretest in all subjects, regardless of the instruction. This improvement was not influenced differently in the groups from percent of failures to reach the correct target since the percent of failures was significantly higher in the pretest compared to the retest across all the groups. Our finding about performance improvement following passive observation corroborates our first hypothesis as well as previous evidence for implicit learning by observation (Heyes and Foster, 2002; Vinter and Perruchet, 2002; Bird and Heyes, 2005; Mattar and Gribble, 2005; Porro et al., 2007). Most interestingly, Mattar and Gribble (2005) found that the subsequent performance of subjects who passively observed a video depicting another person learning to reach in a mechanical environment consisted of a clockwise force field improved to a similar extent as the performance of subjects who were asked to perform an arithmetic addition task that involved both an arithmetic operation and a load on working memory while simultaneously observing the same video clips (Mattar and Gribble, 2005). Also, in another study, even observers who were explicitly told that they did not have to reproduce the observed abduction movements of the right index and middle fingers throughout the training sessions increased the finger abduction force of both their hands (Porro et al., 2007). As opposed to our study, the two studies cited above (Mattar and Gribble, 2005; Porro et al., 2007) also included a control group that did not participate in the observational learning, but only in the tests. The subsequent performance of the AO group improved more significantly than that of the control group, which underscores that improved subsequent performance is related to observational learning rather than the practicing of test performance.

Our finding that both measures, Seq and Delta, improved to a similar extent in passive observers and in observers who engaged simultaneously in MI contradicted our second hypothesis. Only several studies compared the effects of AO+MI vs. AO, and the findings are equivocal (Eaves et al., 2014; Bek et al., 2016; Di Rienzo et al., 2019; Ingram et al., 2019). Similar short-term improvements in maximal isometric strength output of elbow flexor muscles among healthy subjects were found during AO+At and AO+MI (Di Rienzo et al., 2019). Furthermore, the addition of MI to AO did not have a superiority effect compared to MI alone on bicep curl performance in a single case study design (Smith et al., 2019) and on postural sway following a 4-week balance training intervention in healthy subjects (Taube et al., 2014). On the other hand, an “imitation bias” was significantly stronger after healthy subjects had imagined synchronizing a rhythmical action with an observed distractor than after passively observing a rhythmical distractor action (i.e., the participants’ movement responses were biased toward the speed of the previously observed distractor; Eaves et al., 2012, 2014). Similarly, the hemiparetic upper limb motor ability of poststroke subjects was improved more following practice for 4 weeks of MI guided by synchronous AO compared to MI guided by asynchronous AO (Sun et al., 2016). It should be noted, though, that in that study (Sun et al., 2016), only five patients were included in each group. When MI was done after (but not during) the observation of a visual trajectory traced with a cursor, the MI group performed better than the passive observers on novel motor tasks of unfamiliar kinematic trajectories where learning was assessed through changes in the speed–accuracy function across five sessions (Ingram et al., 2019).

Several explanations may be suggested for the equivocal findings. First, it is possible that AO+MI and AO differently affect various parameters of movement. Indeed, Bek et al. (2016) found that movement duration, vertical amplitude, and peak velocity of the participants’ hand movements were significantly closer to the observed action characteristics when instructed to perform AO+MI compared to AO. However, time to peak velocity was closer to the model in the AO group than in the AO+MI group, and no significant effect of instruction was found for horizontal amplitude. Second, the task may affect the necessity to engage in an attention or MI process during AO. For example, the explanation Bek et al. (2016) suggested for the latter nonsignificant effect for the horizontal amplitude is that simply attending to the end point of the movement might have been sufficient to enable replication of the horizontal amplitude, and that attention and MI instruction did not make an additional contribution to accuracy. Given this explanation, it is possible that, since the task in the current study included sequence learning, which could also have been accomplished by following the illuminated target locations, the contribution of MI and attention, as compared to AO, to the response time may have been blurred. It should be noted, however, that learning by means of AO could have led participants to reach toward the next illuminated target more quickly because the direction of the next movement could be anticipated (Brown and Frank, 1987; Hodges and Richardson, 1999). Third, differences in MI instructions may also have a differential effect on behavior measures. For example, in the case of tennis serves, better speed scores and form performances were achieved after using kinesthetic rather than visual representation (Féry and Morizot, 2000; but see also Farahat et al., 2004; Taktek et al., 2008). In the current study, more specific instruction about engaging in kinesthetic MI may have emphasized possible differences between the MI and AO groups. Fourth, nuances of instructions accompanying AO+MI may also affect subsequent performance; for example, imagining during AO (Bek et al., 2016; Di Rienzo et al., 2019) or immediately after AO (Ingram et al., 2019). Similar improvements were found for observers who were instructed to carefully attend to the observed action or to engage in MI during AO in Bek et al. (2016), Di Rienzo et al. (2019), and the current study, which is in line with our third hypothesis. On the other hand, when MI was done after the observation, the participants in the MI group performed better than the observers who were asked to attend to the stimuli in a perceptual control condition, where a visual trajectory traced with a cursor was shown (Ingram et al., 2019). It should be noted, though, that the attention in the latter study (Ingram et al., 2019) was directed to the stimuli (e.g., by being asked to report how many times the cursor changed in a particular direction), whereas the attention in Bek et al. (2016), Di Rienzo et al. (2019), and the current study was directed to the observed human movement. Therefore, the effects of nuances of instructions accompanying AO+MI on subsequent performance still need to be clarified. Fifth, differential instruction for participants does not necessarily assure complete adherence to the intended instruction. Since AO and MI represent internal processing, the possibility cannot be completely ruled out that some passive observers may have engaged in MI or, alternatively, may not have engaged in MI despite being instructed to do so. Sixth, given the relatively large number of trials presenting the same sequence in the learning phase of the current study, it is possible that passive AO was sufficient to achieve sequence learning regardless of instructions. Lastly, simultaneous AO+MI may increase the mental load for some subjects because they have to synchronize their imagined movements with the observed ones. Indeed, in Di Rienzo et al. (2019), subjects perceived the performance of AO+MI as more difficult in comparison to AO alone, and this increased perceived difficulty was associated with autonomic nervous system response patterns attesting to a heightened mental fatigue state.

As there is neurophysiological evidence for significantly greater cortico-motor activity during AO+MI compared to AO alone (Nedelko et al., 2012; Taube et al., 2014; Eaves et al., 2016a; Hardwick et al., 2018), AO+MI has been recommended as a potentially more effective tool for practitioners in motor learning and rehabilitation settings than AO alone (Eaves et al., 2016b). However, when comparing behavioral findings to neurophysiological findings, it should be taken into consideration that increased activity found in neuroimaging studies cannot distinguish between excitatory and inhibitory postsynaptic potentials; therefore, increased activity during AO+MI compared to AO alone cannot directly indicate better behavioral effects in AO+MI than in AO alone.

Our finding about the similar immediate effects of AO+Attention and AO alone (Passive AO) on subsequent performance (see also Mattar and Gribble, 2005) implies that learning processes may function automatically during observational practice. However, there is evidence that the learning processes are influenced by attention during AO (Janelle et al., 2003; Badets et al., 2006; Longo et al., 2008; Gowen et al., 2010; Hayes et al., 2014; Chong et al., 2019). Differences in study protocols may explain the inconsistent results. For example, the chosen measured characteristics of the subsequent performance as well as the aspects of the observed action to whom the attention is directed may explain positive effects on the subsequent performance (Badets et al., 2006; Hayes et al., 2014). Directing attention to the movement trajectory led to a more accurate imitation of timing and spatial position of peak velocity and led to a significant cost in relative timing accuracy (Hayes et al., 2014). Increasing observer attention during AO by explicitly instructing him that he would be required to execute the observed timing task as accurately as possible was more beneficial for learning the movement’s relative timing structure compared to observation without intention to reproduce the task; however, the absolute timing was learned to the same extent by all the observers (Badets et al., 2006). In the current study, the AO+Attention group was instructed to observe the RM sequence carefully and learn it. It is possible that instructing this group to attend to more nuanced aspects of the observed action, such as the trajectory, and measuring those characteristics of the subsequent performance may have yielded differences between observation with and without attentional focusing.

In accord with our fourth hypothesis, AO-related behavioral effects were reflected in both averaged response time (Seq) across all RMs and segmental response time of RM toward a specific direction (Delta) in posttest compared to pretest. This is consistent with previous findings about improved total sequential movement time and segmental intermediate time following AO (Rohbanfard and Proteau, 2011); however, only the Seq, but not the Delta, continued to improve in the retest compared to the posttest. This may point to different consolidation processes underlying general vs. segmental movement time. Instructing the subjects in all the groups to observe the RM sequence, without focusing them to observe a sequence-related segmental movement, may lead to the consolidation of a motor ability that was explicitly experienced before. Such a specific effect is consistent with the notion of specificity (Proteau et al., 1992; Mackrous and Proteau, 2007); an intense practice, with sufficient amount of repetitions, will result in significant gains in performance.

Whereas averaged response time of the RM sequence was improved in posttest compared to pretest to a similar extent, irrespective of the instruction, it continued to improve in the retest (after 24 h) compared to posttest in the Passive AO group only. Similarly, at delayed retention—performed 6 months after explicitly learning a serial reaction time task involving a sequence of foot dorsi and plantar flexions by either MI, verbal rehearsal, or without training—no difference in response times was found among the three groups. This finding also indicates that the effect of some mental practices, with no training, does not necessarily persist over a long period of time (Saimpont et al., 2013). Performance stabilization in the AO+At and AO+At+MI groups and spontaneous performance increase (i.e., off-line learning) in the Passive AO group indicates that the memory representation learned through observation has been consolidated (Robertson et al., 2004; Walker et al., 2005). This finding also suggests that consolidation processes underlying implicit learning (in the Passive AO group) are more efficient following 24 h than in explicit learning (in the AO+MI and AO+At groups), at least in the current setup. In the Passive AO group, the implicit learning most likely relied more on procedural aspects of memory, whereas other groups also relied more on declarative aspects of memory as subjects were consciously trying to identify and learn the sequence. Indeed, declarative and procedural memories are known to recruit common but also different neural networks and to be consolidated differently (Willingham et al., 2002; Walker, 2005; Robertson and Cohen, 2006).

### Limitations

Several caveats of the current study need to be taken into consideration. First, it is possible that some of the subjects, who were asked to imagine the RM sequence, did not actually engage in continuous MI while observing the entire RM sequence, and that the other groups covertly engaged in MI. It is impossible to achieve complete assurance of participant adherence to instructions, especially in studies that include mental practice. On the other hand, if future studies will apply the following suggestions: (1) screen for MI ability; (2) verify, at the end of the session, if the subjects did indeed engage in MI during the entire AO; and (3) inquire about the quality of the imagery, they may be able to potentially refine the effects of instruction on subsequent performance. Second, there is a possibility that during the observation conditions, regardless of instruction, the participants actually had some muscular activity induced by involuntary imitation of the perceived movement despite being instructed to avoid moving and to relax during observation conditions. Using electromyography (EMG) in hand muscles would have ensured this point. However, it should be noted, we found previously that following being instructed to avoid moving during AO, EMG activity in left and right triceps and left and right flexor carpi radialis did not differ between rest and the observation conditions in both healthy subjects (Frenkel-Toledo et al., 2013) and poststroke subjects (Frenkel-Toledo et al., 2014). Third, it is possible that simply being exposed to the physical test at the three time points is enough to drive motor learning over time. Adding a control group that did not observe the RM sequence would have clarified whether the subsequent performance improvement in all the groups was linked to practice-related motor learning, even in the absence of AO, and pointed out the possible differences between subsequent performance improvements, that is, those related to instruction manipulation vs. amount of practice. The rationale for not including such a group is based on previous evidence for learning by observation and the need to achieve our research aim while keeping the number of groups within a reasonable limit. The finding that performance did not differ between groups in pretest ensures at least that all the groups started the experiment with a similar performance. Furthermore, the finding that only in the passive AO group did the average response time improve in the retest as compared to the posttest, while remaining unchanged in the other groups, which performed the same number of RMs in the pretest, posttest, and retest, also supports the delayed effect of instruction manipulation during AO on subsequent performance. Fourth, the subjects performed RMs toward units that were illuminated in the same order as the observed sequence. It is, therefore, possible that the LED illumination helped them perform the RM sequence, and that this may have slightly blurred the differences between the groups. It should be noted, however, that subjects who learned the sequence by means of AO may have been able to reach toward the illuminated LED more quickly because the direction of the next movement could be anticipated. Indeed, changing the expected upper limb movement direction affected limb movement reaction time (Hodges and Richardson, 1999). The preparatory set was manipulated by illuminating the light in the subsequent direction of response with 80% or 20% certainty. Longer movement reaction time was the result of decreased expectation of the required response (Brown and Frank, 1987; Hodges and Richardson, 1999). Last, more focused instruction directing subjects to feel the movement and mentally perceive muscle contractions and stretching might have ensured that the MI group subjects experienced similar kinesthetic imagery, which could potentially have stronger behavioral (Féry and Morizot, 2000; but see also Farahat et al., 2004; Taktek et al., 2008) and neural effects than visual imagery (Stinear et al., 2006; Guillot et al., 2009; Lee et al., 2019). In addition, instructing participants to imagine the RM sequence from an egocentric viewpoint may have also refined the resulting effects because this viewpoint was found to facilitate more efficient imitative behavior than the allocentric viewpoint (Watanabe and Higuchi, 2016). It should be noted that recently, Di Rienzo et al. (2019), who also compared the effects of AO+Attention and AO+MI on subsequent motor performance, addressed some of the current study’s limitations. They specifically included only participants who were screened based on their ability to engage in MI; expressly instructed the non-MI groups to refrain from MI; and directed the MI group to feel the movement during AO; and they did not find a difference in muscle function between the AO+Attention and AO+MI groups. All the limitations noted above should be dealt with in future studies, which may also consider increasing the number of training sessions and including more kinematic measures, such as movement trajectory, to clarify the possible effects of instruction manipulation during AO on additional characteristics of subsequent performance.

### Clinical Implications

Our finding that the performance of the RM sequence improved following either type of instruction corroborates earlier findings, i.e., that AO may be useful as a tool for improving motor abilities in neurological (Ertelt et al., 2007; Pelosin et al., 2010, 2013; Sugg et al., 2015; Patel, 2017; Giorgi et al., 2018; Peng et al., 2019) and orthopedic (Bellelli et al., 2010; Park et al., 2014) conditions. This plus the finding that the performance of the RM sequence continued to improve in the retest for subjects who passively observed it are important because it is very easy to implement this type of instruction during AO training, at least for some patients. For instance, subacute poststroke patients who are hospitalized in a rehabilitation center may have considerable free time to practice, although they may also suffer from fatigue. Passive observation of video clips of relevant motor actions or functions during free time, especially in the critical acute and subacute phases of stroke (Bernhardt et al., 2004), when most spontaneous biological recovery occurs (Duncan et al., 2000; Dromerick et al., 2015), may enhance motor recovery. It should be taken into consideration, however, that patients may respond differently to AO and/or MI, e.g., some poststroke patients may have difficulties engaging in MI due to cognitive impairments (Tong et al., 2017), while others may not benefit from AO if their lesions involve cortical regions that contain large aggregates of mirror neurons [i.e., the IPL and inferior frontal gyrus pars opercularis (IFGpo); for a review, see Rizzolatti et al., 2014]. Also, some poststroke patients with cognitive impairments may perceive the combination of MI+AO as being too difficult, since besides making sure that they have sufficient cognitive abilities to comprehend and carry out MI task instructions, the MI of the motor actions should be coordinated with the observed actions.

## Conclusion

Our results indicate that modulation of top-down aspects of instruction by instructing either to attend to the observed RM sequence or to simultaneously attend and imagine the observed RM sequence does not necessarily have a superiority effect compared to passive AO alone. Whereas performance was improved to a similar extent in the posttest compared to pretest following all instructions, performance continued to improve in the 24-h retention compared to posttest only following passive AO.

## Data Availability Statement

The raw data supporting the conclusions of this article will be made available by the authors, without undue reservation, to any qualified researcher.

## Ethics Statement

The study was approved by the local Ethics Committee of Ariel University.

## Author Contributions

SF-T was involved in planning the experiments as well as data analysis and interpretation and drafting of the manuscript. ZK was involved in planning the experiments, interpretation of data, and drafting of the manuscript. ME was involved in planning the experimental setup and revising of the manuscript. All authors read and approved the final manuscript.

## Conflict of Interest

The authors declare that the research was conducted in the absence of any commercial or financial relationships that could be construed as a potential conflict of interest.
